# Unitarity of Decoherence Implies Possibility of Decoherence-like Dynamics towards Macroscopic Superpositions

**DOI:** 10.3390/e24111546

**Published:** 2022-10-28

**Authors:** Stanislav Filatov, Marcis Auzinsh

**Affiliations:** Department of Physics, University of Latvia, Raina Boulevard 19, LV-1586 Riga, Latvia

**Keywords:** decoherence, quantum superposition, unitarity

## Abstract

Quantum decoherence is crucial to understanding the emergence of the classical world from the underlying quantum reality. Decoherence dynamics are unitary, although they superselect a preferred eigenbasis. Decoherence dynamics result in stable macroscopic, localized, classical-like states. We show that the above-mentioned facts imply the possibility of the existence of decoherence-like dynamics that result in stable macroscopic non-localized non-classical-like states. Being rooted in the fabric of the decoherence theory itself, this property implies environments that steer the decoherence towards, for example, spatial superpositions of macroscopic objects. To demonstrate this, we provide thought-experimental, mathematical and philosophical arguments.

## 1. Introduction

Decoherence (D) theory has been able to show how classical appearances arise from purely quantum substrate. Starting from early insights into einselection [1,2] and ending with the more recent development of quantum Darwinism [3,4,5], it has become the foundation for understanding the quantum-classical transition [6,7,8,9]. Moreover, with its attention to information flows between interacting systems, it has contributed to our understanding of quantum foundations [10,11]. In this work, we will explore the peculiar theoretical consequences of D theory that are built into its construction. First of all, we concentrate on the unitarity of the decoherence process and show that it necessarily means the possibility of decoherence dynamics that do not result in a spatially localized classical-like state. Second, we explore how, following the insights of D theory into the information flows, changes to the properties of particles of the decohering environment might bring about those exotic decoherence dynamics. Questions of transfer of coherence from the superposed object to its environment during the process of decoherence and even the possibility of interference of two different decoherence dynamics are also discussed.

## 2. Analysis

Before the analysis, we should briefly discuss D theory in order to indicate the parts of this theory on which we will base our reasoning. D theory shows how, through strictly unitary dynamics, a set of preferred basis states appears and becomes the stable classical appearance of reality. This is a difficult task because unitary dynamics are not enough for some set of basis states in Hilbert space to be chosen over the other [12,13]. An additional assumption, such as the collapse postulate with the Born rule, is necessary. D is able to do away with collapse and derives the Born rule instead of postulating it. It is able to trace the preferred basis emergence to the nature of the information flow between particles of the system and those of the environment.

Here is a short description of the way the information flows manifest themselves during the process of decoherence. Only states that are able to leave multiple imprints on the environment are the states we are able to perceive [3]. In order to be able to leave an imprint on the environment, there must be symmetry breaking, and some preferred basis must be chosen [14,15]. In particular, which preferred basis will be chosen is traced to the interaction Hamiltonian $H_{se}$ between the particles of environment and the system. It commutes with the preferred basis states. In turn, those commutation relations are rooted in the properties of the particles of the environment (such as the fact that the particles of the environment are localized in space, or that the interaction Hamiltonians are strictly local) [1,6,16,17]. It is this place where our analysis enters.

Toggling the properties of the particles of the environment results in different commutation relations and, therefore, choices of a different preferred basis whose echo propagates through all of the aformentioned reasoning and can result in decoherence towards non-classical-like delocalized macroscopic states. The fact that decoherence dynamics are unitary ensures that the claim of the previous sentence is sound mathematically—we only need to apply the decoherence dymanics in a rotated global basis where the final state is a spatial macroscopic superposition of our choice instead of a localized state. Moreover, the fact that there are more possible initial superpositions of the system than there are final classical-like states (we will make this statement more precise later) ensures that the proposed recipe gives a whole set of dynamics towards a given macroscopic superposition that are not restricted to the simple time-reversal of the decoherence process.

## 3. A Cat in a Box

Let us imagine a gedankenexperiment: a quantum computer where we simulate a box with a cat surrounded by the environment (photons, particles of air). Furthermore, let us assume that it takes as many qubits to simulate the cat in spatial superposition as it takes to simulate the localized cat. Finally, for every run of the simulation, let the cat and the environment initially be unentangled (they may appear in the different corners of the box). Now, we initialize the cat in a macroscopic superposition $\left(HERE+THERE\right)/\sqrt{2}$ and let it decohere by the environment into either HERE or THERE. Then, we initialize the cat in another superposition $\left(HERE+e^{i\theta }THERE\right)/\sqrt{2}$ varying θ every run, letting them all decohere into either of the two localized states. We could even vary superpositions of individual atoms in the cat or vary the states in the environment in order to obtain even more states, all of which eventually result in only two, but for illustrative purposes, it should be enough to note that there are more initial states of the cat than final ones. Certainly, each evolution is unitary, and there is no information loss if we take into account the evolution of the combined environment-system (ES). θ is “absorbed” by the environment, and the initial superposition may be reconstructed, but only if we measure the whole box on some global basis [6,16]. However, when stacked on each other, it looks as though there are many Hilbert space-paths leading to only two states of the cat.

In other words, there is a subspace of the initial environment-system states (all subscripts and superscripts refer to the whole environment-system if not stated otherwise) ${\mathrm{ES}}_{ini}$: ($ES_{ini1},ES_{ini2},ES_{ini3},\ldots ,ES_{iniN}$), a subspace of unitary evolutions of the respective initial states D: ($D_{1},D_{2},D_{3},\ldots ,D_{N}$) and a subspace of the final environment-system states ${\mathrm{ES}}_{fin}$: ($ES_{fin1},ES_{fin2},ES_{fin3},\ldots ,ES_{finN}$). All of those final states may be subdivided into two groups: the cat is HERE, or the cat is THERE.

Unitarity implies that all of the above mentioned states and dynamics may be rotated to obtain a subspace of (different) initial environment-system states ${\mathrm{ES}}_{ini}^{\prime }$: ($ES_{ini1}^{\prime },ES_{ini2}^{\prime },ES_{ini3},\ldots ,ES_{iniN}^{\prime }$), a subspace of unitary evolutions $D^{\prime }$: ($D_{1}^{\prime },D_{2}^{\prime },D_{3}^{\prime },\ldots ,D_{N}^{\prime }$) and a subspace of final environment-system states ${\mathrm{ES}}_{fin}^{\prime }$: ($ES_{fin1}^{\prime },ES_{fin2}^{\prime },ES_{fin3}^{\prime },\ldots ,ES_{finN}^{\prime }$). The rotation may be chosen such that all of those final states may be subdivided into two groups: the cat is $\left(HERE+THERE\right)/\sqrt{2}$, or the cat is $\left(HERE-THERE\right)/\sqrt{2}$. We again obtain multiple dynamics leading to only two ways in which the cat is, and those ways are delocalized superpositions. Decoherence dynamics tend towards non-classical-like states. The recipe for finding those dynamics, initial and final states is simple (theoretically, although not practically). We take ${\mathrm{ES}}_{fin}$ and, recalling that the defining feature of this subspace is that the cat is localized either HERE or THERE, apply a global (to all of the environment-system) rotation to each of the final states such that the cat is now (de-)localized either $\left(HERE+THERE\right)/\sqrt{2}$ or $\left(HERE-THERE\right)/\sqrt{2}$. In this way, we obtain ${\mathrm{ES}}_{fin}^{\prime }$. Let us call this transformation $U_{deloc}$. Applying $U_{deloc}$ to D, we obtain $D^{\prime }$, and to ${\mathrm{ES}}_{ini}$, we obtain ${\mathrm{ES}}_{ini}^{\prime }$.

A few notes are in order before we present an example. First, we purposefully refrain from using the words “state of the cat” or the Bra–Ket notation until now for the final state of the cat and use only capital letters. If we were to say that the final state of the cat is |Here〉 or $|\left(Here+There\right)/\sqrt{2}\rangle$, we would limit the applicability of our argument to the cases when the final environment-system state is separable. Our argument, however, is more general. It applies to any definition of the way in which the cat is localized. We only need the cat to be available in some way, be it through einselection or through gradual consensus of regions of the environment or any other mechanism. As long as the dynamics leading to that localization is unitary (and within the D theory, it always is), the argument works. Second, $U_{deloc}$ is the same for all the states in ${\mathrm{ES}}_{fin}$. It may be said that cat being HERE classically implies a whole set of spatially different locations, not being exactly HERE. For the sake of the clarity of the argument, we avoid such finegraining, but note that it can be implemented and the argument would not change in any substantive way (it would be interesting to think, though, what “not quite $\left(HERE-THERE\right)/\sqrt{2}$” would mean).

Now, let us turn to an example. In order to use the formalism of quantum mechanics and to calculate $U_{deloc}$ explicitly, we have to refer to the final state of the cat. That is why, for this example, we assume that the final state of the environment-system is separable (reminder, the initial state is also separable; see the beginning of this section). We will also use Bra–Ket notation. Let us look at the evolution of the two states in ${\mathrm{ES}}_{ini}$: $ES_{ini1}$ and $ES_{ini2}$. Remember that the initial states in ${\mathrm{ES}}_{ini}$ all contain different spatial superpositions of the cat.

(1) $$\begin{array}{c} D_{1}|ES_{ini1}\rangle =|ES_{fin1}\rangle \end{array}$$

(2) $$\begin{array}{c} D_{2}|ES_{ini2}\rangle =|ES_{fin2}\rangle \end{array}$$

Let us now find $U_{deloc}$ such that it rotates the state of the cat (here and further, we define rotations with respect to what they do to the state of the cat, and the transformation of environment is just the one that is needed for the transformation of the cat to be implemented) in $ES_{fin1}$ (localized cat) onto the state of the cat in $ES_{ini2}$ (delocalized cat). Let us call this new state $ES_{fin1}^{\prime }$ and remember that it differs from $ES_{ini2}$ only by the state of the environment. In other words, let us use the cat that is delocalized in the way of $ES_{ini2}$ as our new final state for the decoherence process. Because the states of the environment-system are separable in the beginning and in the end, we can find $U_{deloc}$ explicitly. It is such a global transformation that transforms the cat state in $ES_{fin1}$ into the cat state in $ES_{ini2}$. The cat states in $ES_{fin1}$ and $ES_{fin2}$ are the same (even if they were orthogonal, one HERE, the other THERE, it would not matter for the argument; the new final state of the cat in $ES_{fin1}^{\prime }$ would still be a delocalized superposition, just orthogonal to the cat state in $ES_{ini2}$ in the same way as HERE is orthogonal to THERE, constituting one of the two possible final cat states for the new decoherence dynamics), therefore, $U_{deloc}$ is $D_{2}^{†}$.

(3) $$\begin{array}{c} D_{2}^{†}|ES_{fin1}\rangle =|ES_{fin1}^{\prime }\rangle \end{array}$$

Now, let us find the initial state $|ES_{ini1}^{\prime }\rangle$ and the decoherence dynamics $D_{1}^{\prime }$

(4) $$\begin{array}{cc} D_{1}^{\prime }|ES_{ini1}^{\prime }\rangle & =|ES_{fin1}^{\prime }\rangle \end{array}$$

(5) $$\begin{array}{cc} D_{2}^{†}D_{1}|ES_{ini1}\rangle & =D_{2}^{†}|ES_{fin1}\rangle \end{array}$$

(6) $$\begin{array}{cc} D_{2}^{†}D_{1}D_{2}D_{2}^{†}|ES_{ini1}\rangle & =D_{2}^{†}|ES_{fin1}\rangle \end{array}$$

Therefore, our $|ES_{ini1}^{\prime }\rangle$ is $D_{2}^{†}|ES_{ini1}\rangle$, and our $D_{1}^{\prime }$ is $D_{2}^{†}D_{1}D_{2}$. We can also apply our newly found $U_{deloc}$ to the second pair of states to find that $|ES_{fin2}^{\prime }\rangle$ is $D_{2}^{†}|ES_{fin2}\rangle =|ES_{ini2}\rangle$; $|ES_{ini2}^{\prime }\rangle$ is $D_{2}^{†}|ES_{ini2}\rangle$, and $D_{2}^{\prime }$ is $D_{2}^{†}D_{2}D_{2}=D_{2}$ (the case of $|ES_{fin2}^{\prime }\rangle$ is interesting. We have chosen the new final state of the cat to be delocalized in the same way as it is delocalized in $|ES_{ini2}\rangle$; the D dynamics towards this state will not stop at it but will continue towards the classical-like $|ES_{fin2}\rangle$ because the environment-system state, and hence, the commutation relations in $|ES_{fin2}^{\prime }\rangle$, are identical with $|ES_{ini2}\rangle$. Although such dynamics are a rarity and are highlighted here due to the simplicity of the example we have chosen to examine, it draws attention to an interesting possibility with ordinary D dynamics: namely, the possibility of and conditions for some classical-like states to not be stable after decoherence but to continue evolving further). Note that the two new final states differ from each other only by the state of the environment there, while the state of the cat is the same in both (particular delocalized superposition).

Finally, applying this transformation to all states in ${\mathrm{ES}}_{ini}$ we obtain a subspace of initial states ${\mathrm{ES}}_{ini}^{\prime }$; applying it to D we obtain decoherence dynamics $D^{\prime }$, and applying it to ${\mathrm{ES}}_{fin}$, we obtain the subspace of final environment-system states ${\mathrm{ES}}_{fin}^{\prime }$, all of which are related in one particular way: the states of the cat there are either of the two orthogonal delocalized superpositions of the $ES_{ini2}$ kind. In fact, as decoherence dynamics are already inscribed in the properties of the particles of the interacting systems, finding a subspace of ${\mathrm{ES}}_{ini}^{\prime }$ is enough for obtaining the decoherence dynamics and the final states automatically.

Hence, we have established that, as far as the conceptual and mathematical framework of D is concerned, the existence of alternative D dynamics leading to exotic superpositions as final states is implicit in D theory.

## 4. Conclusions

In this work, we have shown that if in a theory there exist unitary descriptions of a process that superselects some preferred set of basis states, there must necessarily be other, formally equivalent to the first, processes that superselect other sets of preferred basis states. In the case of decoherence theory, those other preferred basis states are macroscopic spatial superpositions. Here are a few (practical and philosophical) reasons why we consider this result noteworthy. It allows us to think seriously and ask questions about exotic Hilbert-space configurations (which we do in the next section). It uses decoherence theory to show that, although D has been devised to reconcile classical appearances with quantum substrate, in a paradoxical way, it also implies absolutely non-classical appearances. *Finally, this result is also a way to examine quantum theory.* Unitarity and Hilbert-space structures ensure that even apparent information loss is impossible within the quantum theory. If there is a process that results even in apparent loss of information or the preference of some basis of description (in case of D, it is all the possible superpositions of the cat decohering towards either of just two localized possibilities), that loss or preference is compensated for at a different level of the description. A complementary analysis becomes possible within the theory; in the case of D, it is the automatic possibility of similar processes that result in macroscopic spatially delocalized states.

## 5. Discussion

The fact that different dynamics may lead to different preferred final states is an established fact in quantum mechanics. As a reviewer pointed out, in the case of double-well potential, “it is natural that there are environments that can either select the localized basis or the energy eigenstates (delocalized states)”. When applied to decoherence, however, this simple principle is something different. Because D treats the environment as a system and traces the localization dynamics to the internal properties of the particles and not to the global properties of a potential, employing our analysis on D dynamics yields a recipe for microscopic particle-particle interaction rules that lead to delocalizing global dynamics. Delocalizing environments that result from our analysis are not ordinary particle environments superselecting energy eigenstates that happen to be delocalized; rather, they are weird torn-apart space-and-particle environments that superselect delocalized states directly instead of localized ones. In other words, we explore different Hilbert-space configurations from those accessible through models, such as the double-well model. As with new lands, first we can simply tell that they exist and draw attention to them, hoping that later someone will explore them in more detail.

Having established that there might in principle exist environments that superselect spatially delocalized superpositions, it should be noted that judging from our analysis, we must stay agnostic as to what the initial states of the cat are in ${\mathrm{ES}}_{ini}^{\prime }$. Some of them might be localized states; then, we would see a localized cat, when interacting with an exotic environment, decohere into a delocalized cat (a seeming time-reversal), but we cannot say currently whether those states are at all nameable or comprehensible by the classical observer. We also cannot say whether all exotic environment-system states involve non-local interaction Hamiltonians.) Speaking of time-reversal, we have used the word “seeming” because macroscopically, it looks as though it is time-reversal, but it is not a time-reversal of particular D dynamics that led from the superposition to the localization in the first place (“actual” time-reversal). Additionally, judging from the logic of D, where the dynamics are already encoded in the properties of the particles of the environment and the system, the actual time-reversal of particular dynamics is an extremely rare possibility. For it to happen, the localizing commutation relations have to switch to delocalizing exactly by the time when the system was decohered and became localized. That is why, assuming D dynamics contain only dynamics where commutation relations do not change with time, we can say that the actual time-reversal of particular D dynamics is not among the dynamics we have described.

Now let us examine some practical questions that become possible after the possibility of delocalizing macroscopic dynamics is established. The first question, and to us the most interesting, is are there places in the universe that have preference for delocalization or at least do not have a preference for localization? Second, how do we create a mathematical model for microscopic particle-particle interactions that superselect delocalized superpositions? Is it possible to do so without nonlocal Hamiltonians? One avenue we are planning to explore is to take a bit-by-bit measurement model of the system-apparatus-environment from [1] and instead of the apparatus-environment interaction Hamiltonian of Equation (4.2), there take a Hamiltonian that commutes with the superpositional states of an apparatus pointer. This could be the simplest model that superselects the superpositional states of the system. Next, increasing the size of the environment as done in [2] could show how stable these dynamics are, which, judging from the arguments presented in this work, should be as stable as the ordinary localizing dynamics. Third, what about the interaction or interference of two different environments, such as an ordinary and exotic environment? What if their sizes are comparable? Similar questions may be asked about a macroscopic object in a delocalizing environment.

Fifth, how and when did the first symmetry breaking towards localization happen? Although this analysis is not directly related to collapse models such as GRW [18] or Penrose–Diosi [19,20] because it uses the same assumptions decoherence theory does without modifications of current quantum theory, those modifications become important even in the case that decoherence works well for the processes around us now but cannot explain the first symmetry breaking in the past.

Decoherence dynamics are very peculiar. Rooted in the information flows between the interacting parties, it works seemingly automatically. Such dynamics hint at novel ways of manipulating matter and quantum information in general—instead of restricting the conditions so that the system cannot behave in any other way than the desired manner, altering conditions of information exchange so that the system’s evolution is steered towards the desired dynamics, which in this case might even be vaguely defined or unknown.

## Data Availability

Not applicable.

## References

[1] Zurek W. (1981). Pointer basis of quantum apparatus: Into what mixture does the wave packet collapse?. Phys. Rev. D.

[2] Zurek W. (1982). Environment-induced superselection rules. Phys. Rev. D.

[3] Zurek W. (2009). Quantum Darwinism. Nat. Phys..

[4] Blume-Kohout R., Zurek W. (2006). Quantum Darwinism: Entanglement, branches, and the emergent classicality of redundantly stored quantum information. Phys. Rev. A.

[5] Ollivier H., Poulin D., Zurek W.H. (2004). Objective Properties from Subjective Quantum States: Environment as a Witness. Phys. Rev. Lett..

[6] Zurek W. (2018). Quantum Theory of the Classical: Quantum Jumps, Born’s Rule, and Objective Classical Reality via Quantum Darwinism. Phil. Trans. R. Soc. A.

[7] Schlosshauer M. (2005). Decoherence, the measurement problem, and interpretations of quantum mechanics. Rev. Mod. Phys..

[8] Zurek W. (2019). Decoherence of black hole superpositions. Nat. Commun..

[9] Turchette Q.A., Myatt C.J., King B.E., Sackett C.A., Kielpinski D., Itano W.M., Monroe C., Wineland D.J. (2000). Decoherenceand Decay of Motional Quantum States of a Trapped Atom Coupled to Engineered Reservoirs. Phys. Rev. A.

[10] Ollivier H., Poulin D., Zurek W. (2005). Environment as a witness: Selective proliferation of information and emergence of objectivity in a quantum universe. Phys. Rev. A.

[11] Zeh H.D. (2007). The Physical Basis of the Direction of Time.

[12] Hemmo M., Shenker O. (2020). Why the Many-Worlds Interpretation of quantum mechanics needs more than Hilbert space structure. Scientific Challenges to Common Sense Philosophy.

[13] Hemmo T. The Preferred Basis Problem in the Everett Interpretation. https://www.youtube.com/watch?v=WvW0d-gXbqo.

[14] Zurek W. (2007). Quantum origin of quantum jumps: Breaking of unitary symmetry induced by information transfer in the transition from quantum to classical. Phys. Rev. A.

[15] Zurek W.H. (2013). Wave-packet collapse and the core quantum postulates: Discreteness of quantum jumps from unitarity, repeatability, and actionable information. Phys. Rev. A.

[16] Zurek W. Emergence of the Classical from within the Quantum Universe. https://www.youtube.com/watch?v=kEt38oCjQzo.

[17] Zurek W. (2021). Emergence of the Classical from within the Quantum Universe. arXiv.

[18] Ghirardi G.C., Rimini A., Weber T. (1986). Unified dynamics for microscopic and macroscopic systems. Phys. Rev. D.

[19] Penrose R. (1996). On Gravity’s Role in Quantum State Reduction. Gen. Relativ. Gravit..

[20] Diosi L. (1989). Models for universal reduction of macroscopic quantum fluctuations. Phys. Rev. A.

